# Genome Sequences of 20 Bacteriophages Isolated on Gordonia terrae

**DOI:** 10.1128/MRA.01489-19

**Published:** 2020-01-16

**Authors:** Welkin H. Pope, Kristen A. Butela, Rebecca A. Garlena, Deborah Jacobs-Sera, Daniel A. Russell, Marcie H. Warner, University of Pittsburgh SEA-PHAGES, Graham F. Hatfull

**Affiliations:** a Department of Biological Sciences, University of Pittsburgh, Pittsburgh, Pennsylvania, USA; Queens College

## Abstract

We report here the sequences of 20 bacteriophages isolated on Gordonia terrae 3612. These phages span considerable sequence diversity, represent 12 clusters and a singleton genome, and range in genome length from 16.2 kbp to 151.3 kbp. Phages Pupper and SCentae are the first reported Myoviridae phages of Gordonia spp.

## ANNOUNCEMENT

Bacteriophages are the most numerous and diverse biological entities in the biosphere, with an estimated global population of 10^31^ particles, and the population turns over every several days (1). Gordonia terrae is widely distributed in soils, is an opportunistic pathogen in immunocompromised people, and is implicated in nonproductive foaming in wastewater treatment plants (2). To advance our understanding of viral diversity of bacteria in the phylum Actinobacteria, 20 phages of Gordonia terrae 3612 were isolated and sequenced.

Phage isolation was performed either through direct plating of soil filtrate or by enriched growth with G. terrae, as described previously (3). Phages from isolated plaques were purified and amplified, and genomic DNA was extracted using a Wizard DNA prep kit (Promega). Lysates were deposited on carbon-coated copper grids, stained with uranyl acetate, and imaged using an FEI Morgagni transmission electron microscope to determine virion morphology.

Genomic libraries were prepared from phage DNA using an Illumina TruSeq Nano kit. Libraries were sequenced using an Illumina MiSeq instrument, yielding 150-bp single-end reads sufficient to provide enough coverage for each genome (Table 1). Sequence reads were quality controlled and then assembled using Newbler version 2.9; for each genome assembly, a single contig was evaluated for completeness and accuracy using Consed version 29 as described previously (4). Genomic termini were determined using PAUSE (https://cpt.tamu.edu/computer-resources/pause/); for circularly permuted genomes, coordinate number one was chosen by alignment with similar genomes or was immediately upstream of the terminase large subunit gene (4). Genomes were annotated using DNA Master (http://cobamide2.bio.pitt.edu/), ARAGORN (5), tRNAscan-SE (6), Glimmer (7), GeneMark (8), Phamerator (9), BLAST (10), and HHpred (11) and by manual inspection (12). All software was used with default settings.

**TABLE 1:** *Gordonia* phage genometrics

Phage name	Cluster	Genome length (bp)	G+C content (%)	Fold coverage (no. of reads)	Direct plating^*c*^	No. of CDSs^*b*^	No. of tRNAs	Genome ends^*a*^	GenBank accession no.	SRA accession no.
Lozinak	CQ1	93,201	61.9	559	N	180	6	CGCGACGCTC	MF919520	SRX5726432
Toniann	CQ1	92,546	61.9	878	N	180	6	CGCGACGCTC	MF919537	SRX5726440
Patio	CR3	66,251	65.6	3,811	N	90	0	CGCCGCGTAC	MF919542	SRX5726435
BirksAndSocks	CS2	77,354	58.9	1,008	Y	110	1	DTR	MG099940	SRX5726420
Boneham	CS2	77,497	58.9	1,822	N	109	1	DTR	MG757155	SRX5726426
Gorko	CS2	75,158	59	1,064	Y	103	1	DTR	MK801728	SRX5726428
Anamika	CS3	73,851	59.2	907	Y	92	1	DTR	MG099935	SRX5726421
DinoDaryn	CU1	44,936	66.2	1,416	Y	82	0	TCCGGGCCGGTA	KY471269	SRX5726424
Lysidious	CV	50,948	67	874	Y	83	0	TCTCCGGTGA	MF919521	SRX5726433
William	CV	50,678	66.5	456	N	83	0	TCGCCGGTGA	MK801721	SRX5726430
Coeur	CW2	16,223	58	5,134	Y	26	0	ACCCCT	MK801723	SRX5726423
ThankyouJordi	CZ1	53,453	66.4	4,321	N	83	0	CGGCTGGGGA	MK801727	SRX5726439
Bjanes7	CZ2	46,042	66.6	2,827	N	72	0	TACCAGGGGGA	MG099941	SRX5726425
Nordenberg	DE1	57,572	67.3	3,348	Y	84	0	CP	MH976514	SRX5726434
BENtherdunthat	DN	54,867	63.4	1,622	Y	102	0	CTCGGGGCAT	MG099939	SRX5726422
Pupper	DO	150,830	66.1	818	N	234	1	CP	MK977695	SRX5726436
SCentae	DO	151,316	66	665	Y	233	1	CP	MK977696	SRX5726438
Yago84	DR	61,890	70	2,874	Y	83	0	CP	MK801725	SRX5726429
Forza	DS	114,174	53.2	511	Y	164	28	DTR	MK814760	SRX5726427
Reyja	Singleton	41,500	67.4	2,021	N	65	0	TCCGGAGGTA	MK814759	SRX5726437

a Genome ends are 3′ single-stranded overhangs (overhang sequence is listed), direct terminal repeats (DTR), or circularly permuted (CP).

b CDSs, coding sequences.

c Y, yes; N, no.

The 20 phage genomes were assigned cluster and subcluster designations using previously described criteria (3). In brief, genomes with greater than 35% shared gene content with these or previously sequenced actinobacteriophage genomes grouped in the same cluster, and average nucleotide identity values were used to divide clusters into subclusters (3, 13). Of the 20 newly sequenced phages, 13 were assigned into previously designated clusters (Table 1) (3, 14). Six of the 20 phages formed parts of five new clusters (clusters DE, DN, DO, DR, and DS; Table 1), and one phage (Reyja) is a singleton with no close relatives (Table 1). The genomes vary substantially in length from 16 kb (Coeur) to 151 kb (SCentae), with many different genome sizes among them (Table 1), and there is a wide variation in G+C contents (from 53% to 70% in Forza and Yago84, respectively; Table 1). Although most of the 20 phages do not contain any tRNA genes, phage Forza (cluster DS) has 28 tRNA genes, the most for any *Gordonia* phage reported to date (phagesdb.org) (3).

Most of the *Gordonia* phages have siphoviral morphologies. However, phages Pupper and SCentae (cluster DO) are myoviruses with virion morphologies and genome architectures similar to those of the cluster C mycobacteriophages. These are the first known contractile-tailed *Gordonia* phages, but they differ from the cluster C phages—which code for >30 tRNA—in lacking any tRNA genes (phagesdb.org) (13).

### Data availability.

Sequence reads are deposited at NCBI under BioProject accession number PRJNA488469 and SRA accession numbers SRX5726419 to SRX5726440 (Table 1).
